# Innate and adaptive resistance mechanisms to arginine deprivation therapies in sarcoma and other cancers

**DOI:** 10.20517/cdr.2019.49

**Published:** 2019-09-19

**Authors:** Leonard C. Rogers, Brian A. Van Tine

**Affiliations:** ^1^Division of Medical Oncology, Washington University in St. Louis, St. Louis, MO 63110, USA.; ^2^Alvin J Siteman Cancer Center, Washington University School of Medicine, St. Louis, MO 63110, USA.

**Keywords:** PEGylated arginine deiminase, argininosuccinate synthetase 1, resistance, metabolism, arginine

## Abstract

Many cancers lack functional expression of the enzyme argininosuccinate synthetase 1 (ASS1) that is necessary for synthesis of L-arginine. These cancers must import arginine for survival and growth, and this reliance can be targeted by arginine-degrading extracellular enzymatic drugs, most commonly PEGylated arginine deiminase. These enzymes can become targets of the immune system, reducing their effectiveness, but PEGylation improves the *in vivo* stability. Arginine deprivation causes cell death in some cancers, but others gain resistance by expressing ASS1 after a starvation response is induced. Other resistance mechanisms are possible and explored, but these have not been observed specifically in response to arginine deprivation. Future studies, especially focusing on the mechanisms of ASS1 upregulation and metabolic adaptations, may yield insights into preventing or taking advantage of resistance adaptations to make arginine deprivation therapy more effective.

## Introduction

Our understanding of metabolic therapies and how to use them clinically is rapidly evolving. Current therapies based on tumor metabolism are focused on glutaminase inhibitors, isocitrate dehydrogenase inhibitors, pyruvate transport via monocarboxylate transporter 1, and amino acid-degrading enzymes that target asparagine or arginine. It is the ability to systematically degrade arginine and how to apply this clinically that is the focus of this review. Currently, arginine starvation results in an adaptive metabolic reprogramming that renders cells resistant to arginine starvation^[1-3]^. By better understanding the mechanisms of this adaptation, successful therapeutic strategies based on arginine starvation and its related biomarker argininosuccinate synthetase 1 (ASS1) will result.

Amongst the more commonly arginine auxotrophic cancers are all the subtypes of sarcoma. Regardless of histology, about 88% lack significant expression of ASS1^[1]^. This finding is not unique to sarcoma, as most solid tumors, including many melanomas, bladder cancers, prostate cancers, small cell lung cancers, and hepatocellular carcinomas, are deficient in this enzyme^[4-9]^.

Sarcomas often originate in muscle and bone, and these two tissues, along with the heart and lungs, are among the lowest ASS1 mRNA-expressing tissues in the body^[10-12]^. The high incidence of ASS1 silencing in sarcoma may be a consequence of its tissues of origin, but this is a subject of ongoing research. This explanation is also supported by the fact that the *ASS1* gene is rarely mutated or deleted in cancer. Rather, its transcription is regulated by epigenetic modifications and transcription factors^[13-16]^. A common mechanism of *ASS1* silencing is hypermethylation of its promoter region, causing decreased transcription^[13,14,16]^. Some cancers have also shown competition between the repressive HIF-1α and activating c-Myc transcription factors binding to the E-box of the *ASS1* promoter^[15]^. These are all reversible regulation mechanisms that can dynamically respond when the cell is stressed by a lack of arginine. Further work to fully understand the nature of ASS1 silencing in sarcomas is ongoing.

## Urea cycle

**Figure 1:**
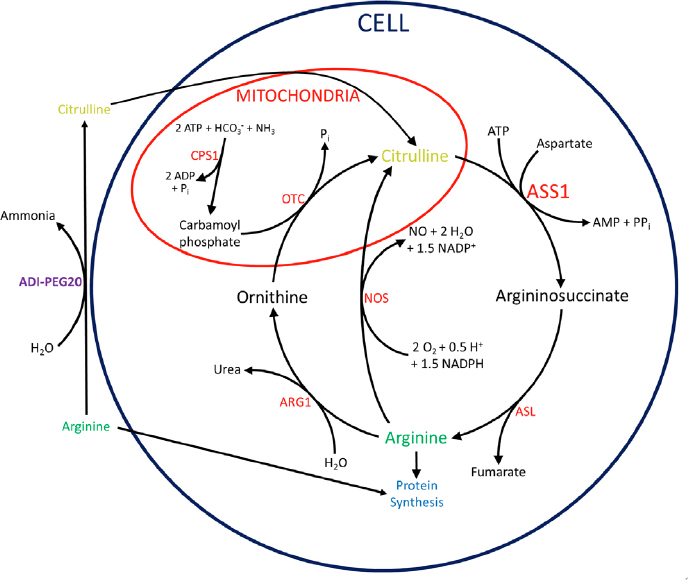
Urea and citrulline-NO cycles with connections to arginine deprivation by ADI-PEG20. Within the mitochondria, CPS1 incorporates ammonia into carbamoyl phosphate. OTC then condenses carbamoyl phosphate with ornithine, forming citrulline. In the cytoplasm, ASS1 catalyzes the condensation of aspartate with citrulline to form argininosuccinate. ASL cleaves argininosuccinate into fumarate and arginine. Arginine is hydrolyzed into ornithine and urea by ARG1, completing the cycle. Alternatively, NOS can hydrolyze arginine to regenerate citrulline while producing NO. ADI-PEG20 degrades arginine to citrulline and ammonia extracellularly. Citrulline can then be imported and converted to arginine by ASS1 and ASL. ADI-PEG20: PEGylated arginine deiminase; CPS1: carbamoyl phosphate synthetase I; OTC: ornithine transcarbamoylase; ASS1: argininosuccinate synthetase 1; ASL: argininosuccinate lyase; ARG1: arginase 1; NOS: nitric oxide synthase; NO: nitric oxide;

The urea cycle in humans, shown in full in Figure 1, takes place mainly in the liver as a way to convert waste ammonia to urea. Ammonia is first converted to carbamoyl phosphate by condensation with carbonic acid and a phosphate group, a reaction catalyzed by carbamoyl phosphate synthetase I^[17]^. The carbamoyl phosphate is then condensed with ornithine with the loss of inorganic phosphate, catalyzed by ornithine transcarbamoylase, forming citrulline^[17]^. ASS1 then catalyzes the condensation of aspartate with citrulline to form argininosuccinate^[17]^. Argininosuccinate lyase (ASL) then cleaves argininosuccinate into fumarate and arginine^[17]^. The arginine is subsequently hydrolyzed into ornithine and urea by arginase 1^[17]^.

Although urea production is its main purpose, the cycle is not isolated and exchanges intermediates with other pathways in the cell. In fact, many cells outside the liver do not perform the full cycle but use portions for other functions, namely to produce nitric oxide (NO) and synthesize arginine, as shown in Figure 1. In adult humans, the kidneys import extracellular citrulline to synthesize arginine for the rest of the body^[18]^. Because of this, most cells in the body have no need to produce arginine, but many still employ part of the urea cycle to produce NO. This is achieved by the enzyme nitric oxide synthase (NOS), using arginine and oxygen to produce NO and citrulline^[19]^. The citrulline can then be recycled to arginine by ASS1 and ASL, in what is sometimes called the citrulline-NO cycle^[19]^.

## Functional consequences of ASS1 silencing

There are many possible benefits of suppressing ASS1. The leading hypothesis is that silencing this gene may accelerate cellular growth by causing an increase in available aspartate^[20]^. Aspartate is consumed in the essentially irreversible reaction catalyzed by ASS1 to ligate citrulline and aspartate [Figure 1]^[17]^. ASS1 deficiency therefore increases levels of aspartate, which is needed to make pyrimidines for nucleotide synthesis, allowing cell proliferation. In fact, the lethal metabolic disorder citrullinemia type I, caused by ASS1 deficiency or mutation, results in increased pyrimidine synthesis and proliferation^[20]^. Supporting this hypothesis are multiple studies that have shown aspartate to be a key limiting metabolite for the growth of cancer cells^[21,22]^.

Another possibility is that ASS1 downregulation helps maintain a higher intracellular pH under acidic stress in the tumor microenvironment^[23]^. This happens when the urea cycle cannot function due to ASS1 loss, leaving basic ammonia free in the cells to scavenge protons. The net consequence of this is increased intracellular pH. Recently, another beneficial role has been proposed for ammonia in cancer, as its nitrogen may be directly incorporated into amino acids to maximize growth efficiency^[24]^. There are likely other beneficial reasons that are yet to be elucidated.

ASS1 deficiency is also predicted to decrease cellular levels of fumarate, a product of the urea cycle downstream of ASS1. Although this metabolite seems to be unexplored in the context of ASS1 deficiency, any decrease is unlikely to be beneficial, considering that excess fumarate has been found to induce epithelial-to-mesenchymal-transition and promote cancer progression^[25]^. ASS1 is also essential in the citrulline-NO cycle that produces NO for signaling^[19]^. However, the citrulline-NO cycle is not necessary, as the substrate needed for NO synthesis is arginine, which can normally be imported from extracellular sources. Regardless of the advantages conferred by ASS1 deficiency, this state renders the cells sensitive to arginine deprivation in a targetable way.

Multiple enzymes are capable of targeting ASS1-deficient cells by degrading arginine in the bloodstream. Arginine decarboxylase (ADC) can be found in bacteria, plants, and mammals^[26]^. ADC converts arginine to agmatine, which is toxic to normal cells and cannot be converted back to arginine, thus limiting the therapeutic potential of this enzyme^[26]^. NOS could also theoretically be used to degrade arginine in the blood, but the increased NO levels may have unintended signaling effects, and NOS has not been used in this fashion to our knowledge. Recombinant human arginase I has been adapted as a drug to degrade extracellular arginine^[26]^. The effectiveness has been greatly improved over time by several modifications, but arginase is less commonly used as a therapy than the following enzyme because of its lower affinity for arginine^[6,26]^. The most widely used of the arginine-degrading enzymes is arginine deiminase, an enzyme found in many microbial organisms that hydrolyzes arginine into citrulline and ammonia^[6,27]^. Arginine deiminase has been conjugated to polyethylene glycol of 20 kDa average size [PEGylated arginine deiminase (ADI-PEG20)], helping to increase its *in vivo* half-life drastically^[28]^.

This drug is being tested in multiple clinical trials and is very promising for multiple reasons. First, it deprives the entire body of arginine regardless of whether the site is accessible, as demonstrated by its efficacy against intracranial glioblastoma, which is protected by the blood brain barrier^[29]^. Importantly, ADI-PEG20 has strong effects on ASS1-deficient tumors while having only benign side effects of injection site rash and increased uric acid levels. These differing effects on normal tissues versus tumors can be at least partially explained by the fact that most cells in the body express ASS1. The liver and kidneys have extremely high levels of the enzyme, as the liver performs the urea cycle for the body, and the kidneys produce excess arginine to export to the blood^[18]^. Most other tissues express varying levels of ASS1 for less clear reasons, but it may be to carry out the citrulline-NO cycle^[19]^. As a result, most tissues can import extracellular citrulline (a product of the ADI-PEG20 reaction) and synthesize arginine for survival. Further explaining the more severe response to ADI-PEG20 in cancer specifically is the increased rate of growth in tumors. While heart, lung, muscle, and bone express very low levels of ASS1, these tissues are relatively static in their growth and therefore unaffected compared to rapidly proliferating cancer cells. When starved of arginine, ASS1-deficient cancers are forced into cytostasis and have been shown to rely partially on autophagy for survival^[1]^. Other methods of arginine acquisition likely also play a part and will be discussed later.

## Resistance

### Adaptive resistance

Some ASS1-deficient cancers undergo cell death when starved of arginine by ADI-PEG20, but many others gain resistance to arginine deprivation^[1-3]^. This is especially common in sarcomas, as they usually do not die as a consequence of arginine starvation^[1,2]^. The most obvious pathway to resistance is upregulation of ASS1 expression. Indeed, this is the only cellular long-term resistance mechanism that has been confirmed to occur in response to ADI-PEG20^[1,6,15,30]^. This path is open to nearly every ASS1-deficient cancer, as mutation or deletion of ASS1 is rare compared to transcriptional silencing. Virtually all regulation of ASS1 has been found at the transcriptional level, and the mechanisms by which ASS1 is upregulated in response to arginine deprivation are the subject of much research.

**Figure 2:**
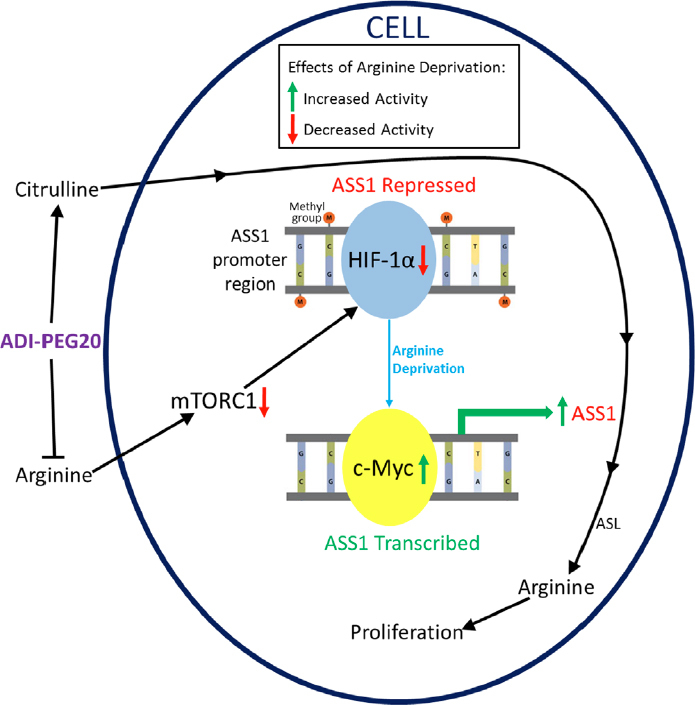
Common Pathway of Resistance to ADI-PEG20. Arginine deprivation caused by ADI-PEG20 inhibits mTORC1, resulting in a decrease of repressive HIF-1α activity. By separate pathways, the positive transcription factor c-Myc is upregulated and causes increased ASS1 transcription and translation. ADI-PEG20 treatment also increases citrulline levels, which the cell can convert to arginine after ASS1 re-expression, resulting in resistance and the ability to proliferate. ADI-PEG20: PEGylated arginine deiminase; ASS1: argininosuccinate synthetase 1; mTOR: mammalian target of rapamycin

Figure 2 illustrates the fully repressed and fully active states of the *ASS1* promoter and shows how arginine deprivation can lead to increased ASS1 expression and resistance to ADI-PEG20. In ASS1-deficient lymphoid malignancies, demethylation of the *ASS1* promoter with a demethylating agent has been shown to rescue cells from ADI-PEG20 treatment^[13]^. More importantly, ASS1-deficient mesothelioma cells were shown to autonomously demethylate their *ASS1* promoter to gain resistance^[31]^. However, more research has focused on the HIF-1α/c-Myc axis. Some cancers display a competition between the repressive HIF-1α and activating c-Myc transcription factors at the E-box of the promoter^[15]^. These two transcription factors seem to be regulated through a multitude of pathways that are affected by arginine deprivation.

Mammalian target of rapamycin (mTOR) activity normally upregulates HIF-1α, repressing ASS1, as shown in Figure 2^[32]^. Because mTOR is a cellular nutrient sensor, its activity is inhibited by the absence of certain amino acids, including arginine. This results in decreased HIF-1α as ADI-PEG20 inhibits mTOR in ASS1-deficient cells [Figure 2]. As HIF-1α levels decrease, the Ras/ERK pathway and phosphoinositide 3-kinase/AKT/GSK-3β kinase cascades are activated, resulting in an increase in c-Myc levels^[15,30]^. Specifically, ERK phosphorylates c-Myc at S62, which is stabilizing^[33]^. Separately, AKT inhibits GSK-3ß from phosphorylating c-Myc at T58, a site that promotes degradation^[33]^. The two pathways thereby work together to stabilize c-Myc as it replaces HIF-1α on the ASS1 promoter. Our understanding of how arginine starvation can be utilized with mTOR pathway modulation needs further exploration.

Another possible mechanism of resistance can result from downregulation of differentiated embryonic chondrocyte 1 (DEC1), another E-box-binding transcription factor, an upstream regulator that promotes an increase in HIF-1α and decrease in c-Myc^[34]^. Silencing DEC1 increased ASS1, lending support to the hypothesis that HIF-1α/c-Myc balance is the dominant mechanism controlling ASS1 expression in many cells^[34]^. However, this study used cells that lacked significant ASS1 promoter methylation in the untreated state, leaving open the possibility that an unmethylated promoter is required for expression^[34]^. Performing a similar study in cells that have been shown to silence ASS1 by promoter methylation would be elucidating.

Arginine starvation can also lead to degradation of HIF-1α by way of the p300-HDAC2-Sin3A chromatin remodeling system^[35]^. The histone acetyltransferase p300 normally maintains specific histone acetylations that help to stabilize HIF-1α on the *ASS1* promoter^[35]^. p300 dissociates upon arginine starvation, and HDAC2 deacetylates these histones, allowing the formation of a HIF-1α-proteasomal complex that leads to degradation^[35]^. Although other transcription factors such as Sp4 and p53 are known to affect ASS1 transcription, there is no evidence that these factors differentially affect ASS1 expression under arginine starvation^[10,15]^.

Upregulation of ASS1 takes time, and cells must sustain themselves in the meantime ultilizing a starvation prosurvival response^[1,2]^. For this, many ASS1-deficient cancers induce autophagy in response to ADI-PEG20^[1,13,36]^. This is caused largely by arginine deprivation inhibiting mTORC1, which allows for increased autophagy. Autophagy, the process by which cells consume parts of themselves, enables the recycling of intracellular arginine, which is incorporated into proteins. This is a short term response. By definition, autophagy is not sustainable indefinitely as a sole source of nutrients. Cells must obtain outside resources to grow. Therefore, autophagy can serve as a sort of bridge to ASS1 upregulation and long-term resistance. However, a reliance on autophagy for any period of time presents a targetable weakness in these cells that can be exploited by combining an autophagy inhibitor with ADI-PEG20 in an attempt to kill cells before they can gain resistance. Indeed, multiple studies have shown that the autophagy inhibitor chloroquine enhances the apoptotic effect of ADI-PEG20^[1,13,36]^. This combination may be more effective in an immune-competent system, as autophagy-defective T cells show enhanced anti-tumor activity^[37]^.

Arginine deprivation also has major metabolic effects on arginine-auxotrophic cells which may contribute to resistance. In one study, melanoma cell lines were found to increase their reliance on both glycolysis and glutamine metabolism after becoming resistant to ADI-PEG20^[3]^. Supporting this conclusion about glutamine is a paper showing that ASS1-depleted cells rely less on extracellular glutamine than ASS1-positive cells, which parallels the difference between cells that are sensitive and resistant to arginine deprivation^[23]^. In addition to the increased reliance on glycolysis in melanoma cells, another study shows a shift toward oxidative phosphorylation in both melanoma and sarcoma cell lines^[2]^. This study once again finds an increased dependence on glutamine metabolism in resistant cells^[2]^. Multiple melanoma and breast cancer cell lines have been shown to become more resistant to glutamine deprivation as ASS1 expression increased in the absence of arginine starvation, whereas loss of ASS1 sensitizes cells to combined arginine and glutamine starvation^[38]^.

Many adaptive changes may be a consequence of increased c-Myc activity, which is necessary for ASS1 upregulation, rather than adaptations to help the cell survive arginine starvation^[3]^. At least one study has demonstrated significant cell death when glutaminase inhibition is combined with ADI-PEG20, signifying that these cells may need glutamine in order to make more glutamate and feed the tricarboxylic acid cycle^[2]^. The upregulation of c-Myc may also allow therapeutic opportunities. Active, nuclear c-Myc is greatly increased by the combination of ADI-PEG20 and docetaxel^[39]^. This in turn leads to increased human equilibrative nucleoside transporter 1 expression, which imports the nucleoside analog gemcitabine, significantly enhancing its efficacy^[39]^.

### Immunogenicity of ADI

Another important aspect of the response to arginine deprivation therapy occurs only *in vivo*. Humans and other mammals mount a strong immune response to pure, recombinant ADI, as it is a foreign bacterial enzyme. This results in a short circulating half-life, about 4 h in mice, and severely limits its effectiveness as a drug^[40]^. This is the main reason that the PEGylated version of the drug was created. The polyethylene glycol cloaks the enzyme from recognition by the immune system and greatly enhance its effectiveness *in vivo*^[28,41]^. However, many patients still have immune responses to ADI-PEG20 over time^[42,43]^. Immune reactions are much slower to occur than with pure ADI, but they can limit the time window in which ADI-PEG20 is effective. Blood arginine levels commonly show a gradual increase after roughly 8 weeks of treatment, which has been correlated to worse patient outcomes when used as a single agent^[42-44]^. However, this window of effectiveness can be extended to at least 18 weeks by some combination therapies, as has been shown recently in multiple clinical trials utilizing triple drug regimens that include ADI-PEG20^[45,46]^. Correlated with this was a delayed buildup of anti-ADI-PEG20 antibodies^[42,44-46]^. These promising results should encourage further research to develop combination therapies that kill quickly. Additionally, the immune adaptation period can likely be harnessed for therapeutic purposes as tumors metabolically evolve in that time *in vivo*.

While the immune system can neutralize ADI-PEG20, the drug may also have a negative effect on the immune system by depleting arginine. Low arginine levels are known to inhibit T cell proliferation, as myeloid-derived suppressor cells do this naturally through the activity of arginase I^[47]^. An arginase inhibitor therefore has potential to be effective in combination with ADI-PEG20 if anti-tumor T cells express ASS1 themselves and can make arginine. However, this may inadvertently also provide tumor cells with arginine, possibly negating the immune benefit. Arginine depletion has another detrimental effect on the anti-tumor activity of the immune system, as ASS1 upregulation causes increased programmed death-ligand 1 (PD-L1) expression, which is a negative regulator of T cells^[48,49]^. This suggests PD-1/PD-L1 immune checkpoint blockade to be used in conjunction with ADI-PEG20^[48,49]^. There is even evidence that ADI-PEG20 can enhance infiltration of T cells into tumors in mice, giving further support to the potential of this combination^[49]^.

ASS1-deficient cells have also been found to have higher levels of cationic amino acid transporter 1 (CAT-1), which is the most important arginine importer and a potential drug target^[50,51]^. Furthermore, ASS1-deficient cells have been shown to increase import of arginine through CAT-1 in response to ADI-PEG20 treatment^[51]^. The extent of the contribution of this response to resistance was not determined, but it is likely mild and not enough to sustain growth, since extracellular arginine levels remain extremely low.

### Other mechanisms of resistance

ASS1 re-expression and autophagy have been discussed, but there are a plethora of other ways for cells to obtain arginine, many of them unexplored as pathways for arginine deprivation resistance. Here we summarize what is known about such pathways and their relevance to ADI-PEG20 resistance. Macropinocytosis can be utilized by cancer cells to import and break down extracellular proteins for any amino acid, including arginine^[52,53]^. A reliance on this pathway is common in Ras-transformed cells and pancreatic cancers, but can also be used by other cancers^[52,53]^. It is therefore possible that macropinocytosis may be an important resistance pathway to arginine deprivation, as it has been shown to increase when mTORC1 is inhibited^[54]^. Similarly, many breast cancers can import necrotic cell debris in a process called necrocytosis^[55]^. After three days of amino acid starvation, 35%-71% of cellular protein biomass was found to originate from necrocytosed peptides, and this process also provides carbohydrates, lipids, and nucleotides^[55]^. Macropinocytosis and necrocytosis can also contribute significant amounts of amino acids under normal growth conditions, suggesting that these pathways could immediately provide relief from arginine starvation even without ASS1 upregulation^[52,53,55]^. There is also some evidence that extracellular proteins, particularly albumin, can be taken up by receptor-mediated endocytosis, although this process has not been shown to promote cell growth to the same extent as macropinocytosis^[54,55]^.

Phagocytosis is another possible pathway to resistance, albeit unlikely, as only a few cell types perform this activity. With this method, the cell would gain both cytoplasmic arginine and protein-incorporated arginine from consumed cells, exosomes, or other material, along with all other nutrients necessary for survival in the case of whole-cell phagocytosis. This phenomenon has rarely been demonstrated in nonhematopoietic cells, but some breast cancer cell lines are able to phagocytize yeast and extracellular matrix^[56,57]^. Some cancers can also perform a process called entosis, whereby one cell invades another^[58]^. The internal cell can then either be released or degraded by lysosomal enzymes, providing nutrients to the engulfing cell^[58]^. The end result would be almost identical to phagocytosis with regards to nutrients. It is also conceivable that slower-adapting cells are engulfed more often, which would hasten the onset of resistance in the tumor overall.

Direct sharing of arginine from other cells through gap junctions (for example, immune cells and fibroblasts in the stroma) also has not been ruled out. Heterologous gap junctions are presumably rare but have been found between some cancer and non-cancer cells^[59,60]^. However, gap junctions are generally downregulated in cancer and therefore less likely to play a role in resistance^[61]^. Alternatively, other cells in the body may supply arginine to ASS1-deficient cancer cells indirectly. This mechanism has been shown with another amino acid, as mesenchymal cells in the microenvironment where leukemic cells grow can produce and secrete asparagine, conferring resistance to asparaginase treatment in some cases of acute lymphoblastic leukemia^[62]^. The most effective form of resistance to arginine depletion is innate. Many cancers do not downregulate ASS1 and are therefore immune to arginine deprivation therapy. Some apparently ASS1-deficient cancers may also be heterogeneous in their expression. It would take only a small number of highly expressing cells to eliminate the possibly of eradication through arginine deprivation treatment. Even if the other cells all died, ASS1-high cells would be clonally selected and grow out. This heterogeneity has not been a focus of research, but the field would benefit from its investigation.

## Current clinical trials

There have been many completed clinical trials involving ADI-PEG20, while others are currently active or planned. These trials are summarized in Table 1. Numerous cancer types are included across the trials, and many involve ADI-PEG20 monotherapy. However, some test ADI-PEG20 in combination with other drugs, which is likely to be more effective for many cancers that are not killed by monotherapy. There is great potential for similar combination trials in the future, as ADI-PEG20 causes many potentially targetable adaptations in ASS1-deficient cancers while having few side effects.

**Table 1:** List of all clinical trials of ADI-PEG20

NCT Number	Conditions	Drugs	Status
NCT03254732	Advanced Solid Cancers	ADI-PEG20|Pembrolizumab	Recruiting
NCT02029690	Pleural Mesothelioma Malignant Advanced |Peritoneal Mesothelioma Malignant Advanced|Non-squamous Non-small Cell Lung Carcinoma | Uveal Melanoma | Hepatocellular Carcinoma | Glioma | Sarcomatoid Carcinoma	ADI-PEG20	Active
NCT03449901	Soft Tissue Sarcoma	ADI-PEG20|Gemcitabine |Docetaxel	Recruiting
NCT02101580	Advanced Pancreatic Cancer	ADI-PEG20	Active
NCT01287585	Hepatocellular Carcinoma	ADI-PEG20	Completed
NCT01497925	Solid Tumors | Prostate Cancer	ADI-PEG20	Completed
NCT01948843	HER2 Negative Metastatic Breast Cancer	ADI-PEG20	Completed
NCT02101593	Hepatocellular Carcinoma	ADI-PEG20	Completed
NCT01266018	Small Cell Lung Cancer	ADI-PEG20	Terminated
NCT01910012	Acute Myeloid Leukemia	ADI-PEG20	Active
NCT02102022	Advanced Gastrointestinal(Gl) Malignancies|Hepatocellular Carcinoma| Gastric Cancer| Colorectal Cancer	ADI-PEG20 plus modified FOLFOX6	Recruiting
NCT01279967	Malignant Pleural Mesothelioma	ADI-PEG20	Unknown status
NCT02875093	Acute Myeloid Leukemia	ADI-PEG20| Cytarabine	Recruiting
NCT01910025	Non-Hodgkin’s Lymphoma	ADI-PEG20	Completed
NCT02709512	Mesothelioma	ADI-PEG20| Pemetrexed|Cisplatin	Recruiting
NCT01665183	Cutaneous Melanoma, Uveal Melanoma, Ovarian Carcinoma or Other Advanced Solid Tumors	ADI-PEG20	Completed
NCT01528384	Argininosuccinate Synthetase-Deficient Cancers	ADI-PEG20	Completed
NCT03498222	Carcinoma, Non-Small-Cell Lung	Atezolizumab|Pemetrexed|Carboplatin| ADI-PEG20	Not yet recruiting
NCT02006030	Unresectable Hepatocellular Carcinoma	ADI-PEG20	Completed
NCT00520299	Metastatic Melanoma | skin Cancer| Neoplasm	ADI-PEG20	Completed
NCT00450372	Melanoma(Skin)	ADI-PEG20	Completed
NCT03922880	Uveal Melanoma	ADI-PEG20| Nivolumab|lpilimumab	Recruiting
NCT00056992	Carcinoma, Hepatocellular	ADI-PEG20	Completed
NCT00029900	Melanoma | Neoplasm Metastasis	ADI-PEG20	Completed

ADI-PEG20: PEGylated arginine deiminase

## Conclusions

Arginine auxotrophic cancers can be targeted by enzymes that degrade arginine in the blood. However, many cancers gain resistance to the most widely used of these enzymes, ADI-PEG20, by upregulating ASS1 and converting the degradation product citrulline back into arginine. Before ASS1 levels are sufficiently increased for resistance, many cancers rely on autophagy to temporarily recycle arginine and sustain themselves. There are also other possible mechanisms by which cells could gain resistance. The most studied of these is macropinocytosis, with which cells can ingest extracellular proteins to use as amino acids sources. Additionally, some parts of a heterogeneous tumor may express higher levels of ASS1 than the rest and be resistant from the start of treatment. Cells that are sensitive to arginine deprivation respond with numerous changes in cellular metabolism and transcription factor activity, amongst other things. Many of these changes can be targeted by other drugs in combination with ADI-PEG20. Exploration of the multitude of metabolic adaptations to arginine deprivation has only just begun, and the future of therapy development is bright.

## Declarations

### Copyright

© The Author(s) 2019.
